# Topical combined tranexamic acid and epinephrine versus topical epinephrine in control of intraoperative bleeding of external dacryocystorhinostomy

**DOI:** 10.1007/s10792-023-02789-w

**Published:** 2023-07-15

**Authors:** Moustafa A. Salamah, Hani A. Al Bialy, Marwa A. Khairy, Ali Goda Ali

**Affiliations:** Department of Ophthalmology, Faculty of Medicine, Zagazig University, Zagazig, Egypt

**Keywords:** Dacryocystorhinostomy, Bleeding, Tranexamic acid, Epinephrine

## Abstract

**Purpose:**

To compare the efficacy of gauze soaked with combined tranexamic acid (TXA) (100 mg/ml) epinephrine 1:200,000 versus gauze soaked with only epinephrine 1:200,000 used to guard against intraoperative bleeding in external Dacryocystorhinostomy (DCR).

**Patients and methods:**

The study included 33 patients; only 30 patients fulfilled the inclusion criteria and were divided randomly into 2 groups using the random numbers table, with 15 patients in each group. The first group (Group A) was operated upon using gauze soaked with combined TXA (100 mg/ml) and epinephrine 1:200,000, while the second group (Group B) was operated upon using gauze soaked only with epinephrine 1:200,000.

**Results:**

The amount of bleeding was significantly lower in group A (29.4 ± 17.1 ml) compared to group B (49.1 ± 18.1 ml), with a *P* value = 0.005. In addition, the number of used gauzes and total surgical time was significantly lower in group A compared to group B, with *P* value = 0.008 and 0.01 respectively.

**Conclusion:**

External DCR using gauze soaked with combined TXA (100 mg/ml) and epinephrine 1:200,000 showed a significant reduction in the amount of intraoperative bleeding compared to gauze soaked with epinephrine 1:200,000 only. The reduction in the amount of bleeding with the addition of TXA resulted in clearer surgical field, shorter surgical time and more surgeon satisfaction.Query

## Introduction

Dacryocystorhinostomy (DCR) is a relatively common procedure performed by oculoplastic surgeons to create an anastomosis between the lacrimal sac mucosa and the nasal mucosa to bypass nasolacrimal duct (NLD) obstruction. External approach DCR remains the gold standard despite the wide use of the endoscopic approach [1].

Control of intraoperative bleeding during DCR is of utmost importance as bleeding may obscure the already narrow operative field, making recognition of the sac wall or nasal mucosa very difficult [2].

The amount of bleeding during external DCR varied in different studies but was reported between 3 and 25 ml [2–5]. The nasal mucosa was the most common source of bleeding in DCR surgery (76.1%), followed by the angular vein (13.6%), bones (6%), and muscles (4.3%) [6].

Many approaches have been adapted to avoid such complication, including pre-operative careful patient preparation, controlling blood pressure, ruling out blood dyscrasias, discontinuing anticoagulant and antiplatelets drug intake. Also, intraoperative use of epinephrine along with local anesthetics unless contraindicated by medical cause, appropriate surgical technique to avoid known blood vessels with judicious use of cautery and raising the head end of the surgical table [7].

TXA (trans-4-aminomethyl cyclohexane carboxylic acid) is a synthetic lysine analogue that prevents breakdown of the blood clot by reversibly blocking the binding sites of plasminogen and prevents plasminogen activation to plasmin and the lysis of polymerized fibrin in the blood clot. Because of its hemostatic activity, wide availability, and limited side effects, it has also been widely studied for the prevention and treatment of haemorrhage in trauma and several types of elective surgery [8].

TXA is used topically during tooth extraction, orthopedic procedures, cardiac surgery, and many other surgical procedures [9]. To our knowledge, this is the first study to investigate the use of topical TXA in external DCR.

The aim of this study is to compare the efficacy of gauze soaked with combined TXA (100 mg/ml) and epinephrine 1:200,000 versus using gauze soaked only with epinephrine 1:200,000 in intraoperative bleeding control in external Dacryocystorhinostomy.

## Patients and methods

### Study design

This is a single-center, double-blind, prospective, randomized, clinical study. The study was conducted in the Ophthalmology Department, Zagazig Faculty of Medicine during the period between March 2022 and September 2022 to treat patients with epiphora caused by primary acquired nasolacrimal duct obstruction with or without + ve regurge test.

This research was approved by the Institutional Review Board of Zagazig University Faculty of Medicine (IRB#9231-27-3-2022) and was adherent to the ethical principles outlined in the Declaration of Helsinki as amended in 2013. Also, approval of this study design was obtained from the Pan African Clinical Trial Registry (ID number PACTR202206674510595). https://pactr.samrc.ac.za/

### Participants

Patients aged more than sixteen years with primary acquired nasolacrimal duct obstruction with or without a positive regurge test were included in this study.

Patients with uncontrolled hypertension, known TXA allergy, personal or familial history of bleeding disorder, or on anticoagulant therapy, the diagnosis of coexisting nasal pathologies that could influence the outcome of the surgery, and patients with a history of trauma or laceration to the lacrimal passages were excluded from this study.

The surgical technique, likely post-treatment results and potential complications were explained to all patients. Written consent was obtained from all patients. Consent includes permission to publish their photos.

Finding that mean ± SD of the amount of bleeding after 60 min according to Hamed and Hamed [10] among group A (TXA group) was 6.7 ± 4.7 and that among group B (epinephrine group) was 11.1 ± 3.6, so sample size was calculated by openEpi program to be 30 eyes [15 eyes in each group] with confidence level of 95% and power of test 80%

**Fig. 1:**
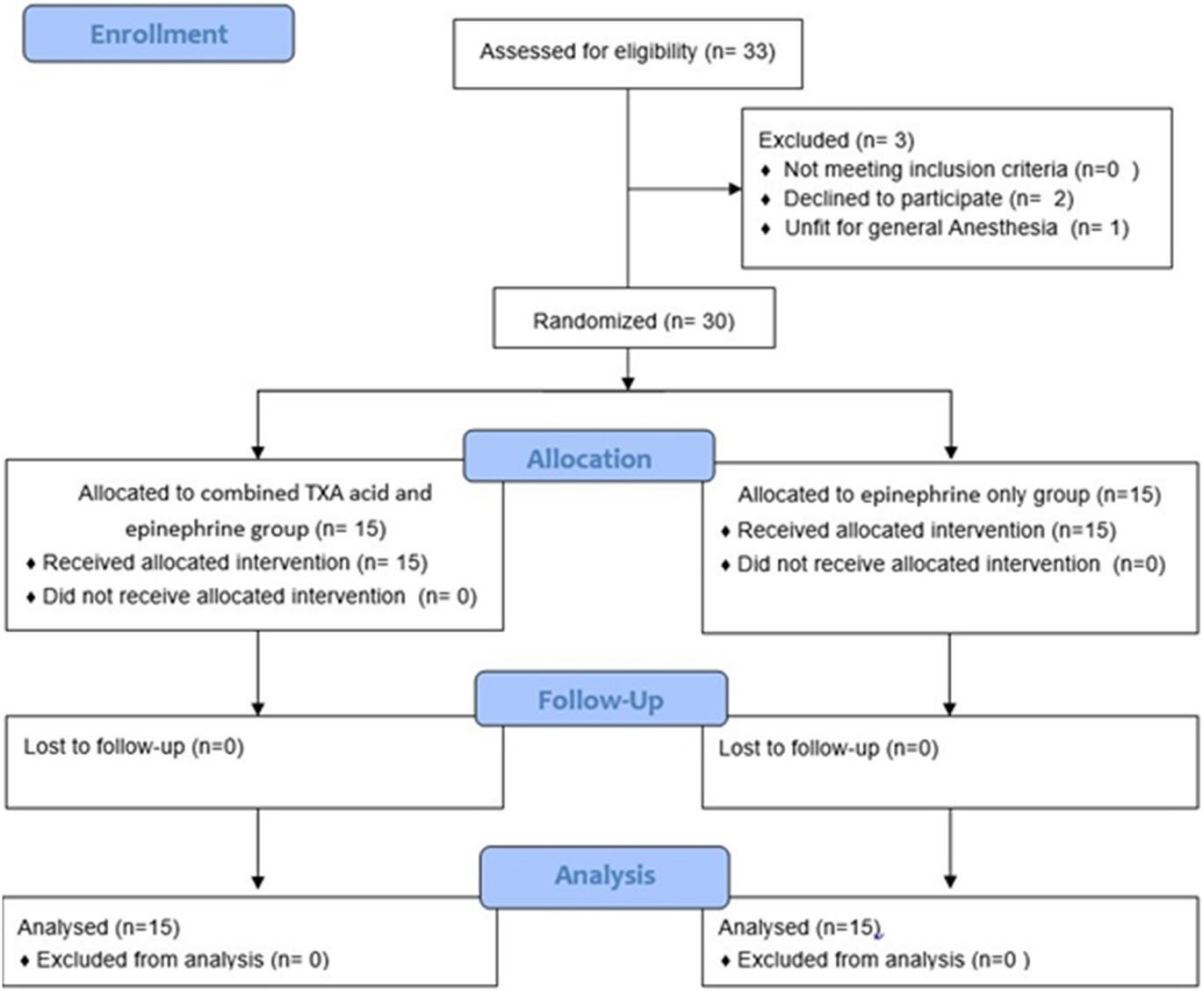
CONSORT flow diagram

The study included 33 patients with primary acquired nasolacrimal duct obstruction. 2 patients refused to sign the consent and didn’t participate in the study, and one patient didn’t fit for general anesthesia. Those patients were excluded from the study. 30 patients were divided randomly into 2 groups using the random numbers table, with 15 patients in each group. The first group (Group A) was operated upon using gauze soaked with combined TXA (100 mg/ml) and epinephrine 1:200,000, while the second group (Group B) was operated upon using gauze soaked only with epinephrine 1: 200,000. A CONSORT flow diagram is shown in (Fig. 1).

### Preoperative assessment

All patients were subjected to preoperative assessment including full ophthalmological examination, assessment of epiphora by fluorescein dye disappearance test, assessment of the patency of the lacrimal system by probing and syringing; in addition to ENT examination and laboratory investigations including bleeding and clotting times.

### Surgical technique

All surgeries were performed under general anesthesia by a single surgeon after nasal packing of oxymetazoline 0.025%.

**Fig. 2:**
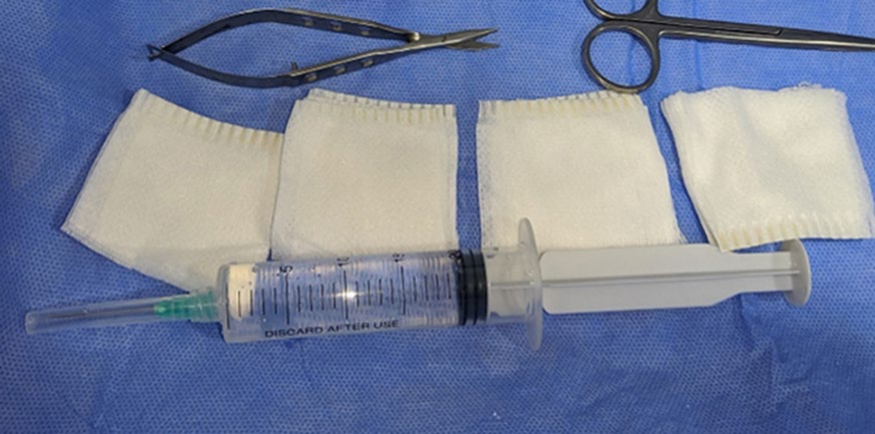
a 20 mL syringe containing either combined tranexamic acid (100 mg/ml) and epinephrine 1:200,000 or epinephrine 1:200,000 alone, as well as fixed size gauze

The different mixtures were prepared in coded 20 mL syringes before surgery. The coding list was opened only after the completion of the study. The surgeon and the patient did not know the type of the mixture that was used (Fig. 2).

**Fig. 3:**
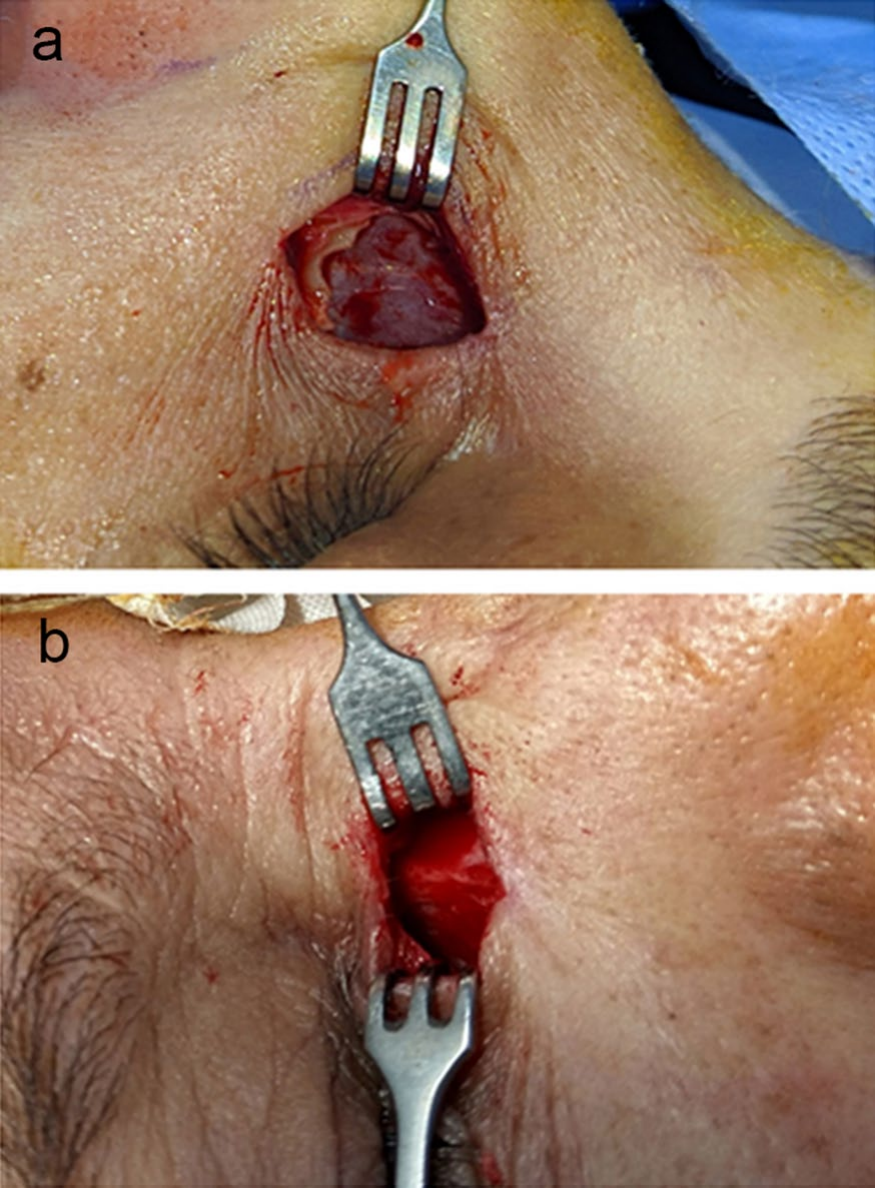
Surgical field in group A **a**, versus group B **b**, with less bleeding, better visualization and demarcation of anatomical structures

In group A, soaked gauzes (standard size 4X4 cm) of combined TXA (100 mg/ml) and epinephrine 1:200,000 were used to control bleeding. A time of two minutes was fixed for each application of soaked gauze (Fig. 3a).

External DCR was performed with the standard technique. A skin incision was performed and blunt dissection to the periosteum overlying the anterior lacrimal crest was made. The periosteum was then incised and elevated. Osteotomy was made approximately 10 mm in front of the anterior lacrimal crest and inferiorly to expose the upper part of the nasolacrimal duct. A large flap was performed, a long vertical top to bottom incision was made with blade No. 11 on the medial sac wall. A vertical long top to bottom incision with the blade was made on the nasal mucosa opposite to that of the sac. Both the upper and the lower puncta were dilated with a punctal dilator, then each limb of the bicanalicular Crawford silicone stent was passed through the corresponding canaliculus and were drawn out of the nose. Both the lacrimal sac and the nasal mucosal flaps were sutured using a 5/0 vicryl suture. Finally, the orbicularis was sutured back with 6-0 vicryl followed by skin with 6-0 silk.

In group B, the same procedure was performed except for the application of soaked gauze of only epinephrine 1:200,000. A time of two minutes was fixed for each application of soaked gauze (Fig. 3b).

The amount of blood loss in each case was measured at the end of the DCR operation from the suction bowel and calculated using a large 50 cc syringe. To determine the amount of bleeding, the amount of saline utilized for irrigation is deducted from the overall amount of bleeding. The duration of surgery was calculated by registering the time of operation start and end.

### Follow up of patients

All patients were examined on the first postoperative day. The nasal pack, if any, was gently removed and hemostasis was assessed. The wound was cleaned with 5% betadine, and the patient was discharged on oral Amoxycillin/clavulanate (Hibiotic) one-gram tablets (Amoun pharmaceuticals company, Egypt) twice daily, ibuprofen (Brufen) 400 mg tablets (Abbot, Egypt) three times daily, and topical Antibiotic Gatifloxacin (Tymer) (Jamjoom Pharmaceuticals, Jeddah, Saudi Arabia) eye drops four times daily for 10 days.

One week postoperative, the skin sutures were removed, and oral medications discontinued. The patient was reviewed at 6 and 12 weeks. Tube removal was usually done at 12 weeks.

### Outcome Measures

Our primary outcome measure was to compare the amount of intraoperative bleeding (measured in ml) during external DCR using gauze soaked with combined TXA (100 mg/ml) and epinephrine 1:200,000 versus using gauze soaked only with epinephrine 1:200,000.

Secondary outcome measures were to report the number of used gauzes in each group, measure surgical time, and assess the degree of surgeon satisfaction regarding the clarity of the surgical field and the ease of the surgery.

### Statistical analysis

Statistical analysis of this study included 30 patients; with 15 patients in each group. Data were collected through patient history taking, examination, recording of intraoperative events including the amount of intraoperative blood loss, the number of used gauzes, the total surgical time, and the degree of surgeon’s satisfaction as well as intra and postoperative complications. Qualitative variables were represented as numbers and percentages, while continuous quantitative values were expressed as mean ± standard deviation (SD). Pearson’s Chi-square (X2) test, Paired sample T test and the ANOVA test were used in statistical analysis, a *p* value < 0.05 was considered statistically significant. The data were coded and analyzed using the Statistical Package for the Social Sciences (SPSS) V16 software.

## Results

Thirty patients were included in this study; 15 patients in each group without a statistically significant difference in terms of age and sex between the two groups. (Table1).

**Table 1:** Demographic characteristics of the trial participants

Variable	Group A (N = 15)	Group B (N = 15)	Test	*P* value
Age				
Mean ± SD	39.9 ± 11.5	4.2 ± 14.4	1.198#	0.241
Range	18–56	17–67		(NS)
Sex				
Female	8 (53.3%)	10 (66.7%)	0.556$	0.456
Male	7 (46.7%)	5 (33.3%)		(NS)

#: t test. $: Chi-square test. NS: non-significant (*p* > 0.05)

Intraoperative bleeding (measured in ml), number of used gauzes, and the total surgical time were significantly lower in group A compared to group B. (Table 2).

**Table 2:** Clinical characteristics of the studied groups

Variable	Group A (N = 15)	Group B (N = 15)	Test	*P* value
Bleeding amount (ml)				
Mean ± SD	29.4 ± 17.1	49.1 ± 18.1	− 3.046	0.005
Range	12–72	24–88	#	(S)
Number of used gauzes				
Mean ± SD	2.4 ± 1.1	4.2 ± 2	− 2.879	0.008
Range	1–5	1–8	#	(S)
Operation time (minutes)				
Mean ± SD	36 ± 8.7	46.1 ± 11.7	− 2.651	0.01
Range	28–54	28–68	#	(S)
Clarity of field				
Not clear	1 (6.7%)	4 (26.7%)		
Clear	8 (53.3%)	7 (46.7%)	8.311	0.081
Very clear	6 (40%)	4 (26.7%)	$	(NS)
Satisfaction				
No	3 (20%)	3 (20%)		
Mild	2 (13.3%)	7 (46.7%)		
Moderate	2 (13.3%)	3 (20%)	6.578	0.087
High	8 (53.3%)	2 (13.3%)	$	(NS)

#: t-test. $: Chi-square test. NS: non-significant (*p* > 0.05). S: significant difference (*p* < 0.05)

Surgeon satisfaction regarding the clarity of the surgical field and the ease of surgery was compared between the two groups and graded into four grades. Although a clearer surgical field and more surgeon satisfaction were observed in group A compared to group B, statistical analysis showed no significant difference between the two groups. (Table 2).

Further statistical analysis was done using the ANOVA test to detect the relationship between the surgeon’s satisfaction and intraoperative parameters. The analysis showed that the lower the amount of intraoperative bleeding measured in ml is associated with fewer number of gauzes used and clearer surgical field, and all these parameters are associated with higher surgeon satisfaction and shorter surgical time.

## Discussion

Minimizing bleeding during external DCR is an important goal as bleeding can obscure the operative field especially the lacrimal sac and the nasal mucosa; and subsequently increase the DCR failure rate. Epinephrine has been widely used to reduce the per operative bleeding in many surgeries via its vasoconstrictor effect. Epinephrine also has a procoagulant effect by increasing the platelet aggregation via its alpha-adrenergic effect [11]. The vasoconstrictor effect of the epinephrine varies according to the vessel type whether arteries, arterioles, precapillary sphincters, capillaries, venules, and veins [12]. However, delayed intraoperative bleeding may occur when the vasoconstrictive effect had wane leading to rebound bleeding by several mechanisms including local hypoxia of the tissues and to the acidosis caused by the prolonged vasoconstriction and β adrenergic effect causing rebound hyperemia [13]. Therefore, addition of antifibrinolytic agent as TXA has been proposed, aiming to maintain the already formed blood clot.

Local application of TXA has been investigated in many surgical interventions to reduce intraoperative bleeding and maintain a dry surgical field with subsequently less surgical time. This addition proved its efficacy in facelift surgery [14], joint replacement [15], minor oral surgeries [16], as well as many dermatological procedures like Mohs micrographic surgery [17].

In this study, we investigated the efficacy of adding TXA to epinephrine-soaked gauze in reducing the total amount of bleeding in external DCR, aiming for the best bloodless intraoperative field during the procedure and reducing the total operative time. To our knowledge, this is the first study to investigate the use of topical TXA in external DCR.

Agrwalla and Dora in 2016 assessed the efficacy of systemic administration of either oral TXA 500 mg tablets, ethamsylate tablets or botropase injection preoperatively versus placebo (Vitamin B complex) in reducing the amount of intraoperative bleeding and shortening the surgical time during lacrimal sac surgery. The authors found a significant difference in the mean operating time between the TXA group and the placebo group (30 min and 56 min, respectively). Also, the number of gauge pellets soaked with blood postoperatively was calculated and was significantly lower in the TXA group (22 pellets) versus the placebo group (38 pellets). [18] Our results were comparable with those in this study. The amount of intraoperative bleeding was significantly lower in group A (29.4 ± 17.1 ml) as compared to group B (49.1 ± 18.1 ml). The mean operative time was 36 min in group A and 46.1 min in group B, and a significantly lower number of gauzes were used in group A compared to group B (2.4 ± 1.1 and 4.2 ± 2 respectively, with *p* value = 0.008). However, we think that the topical route of TXA administration may be more convenient than oral administration to avoid any systemic hazards as GIT complications, hypersensitivity reaction and the rare thromboembolic events [18].

Our results nearly matched those of Caesar and McNab, who reported a mean time of 36 min after use of a mixture of local anaesthetic composed of a 1:1 mixture of 2% lidocaine with 1:100,000 epinephrine and bupivacaine with 1:100,000 epinephrine. Caesar and McNab also reported a mean blood loss of 4.5 mL, while in our study we reported a mean blood loss of 29.4 ± 17.1 ml in group A and 49.1 ± 18.1 ml in group B. The authors themselves stated that the reported blood losses in various studies have varied from a mean of 6.3 to 250 ml due to variable techniques used in measuring blood loss [3].

In our study surgeon satisfaction regarding the clarity of the surgical field and the ease of surgery was compared between the two groups and graded into four grades. Although a clearer surgical field and more surgeon satisfaction were observed in group A compared to group B, statistical analysis showed no significant difference between the two groups. This may be attributed to low number in each group in relation to high number of grades of satisfaction. However, no difference in final clinical outcome over the follow-up visits was noticed.

The results of this study suggest that the roles of epinephrine and TXA are complementary and the short time of action of the epinephrine could be avoided with the clot stabilization effect of TXA. This effect is supported by the laboratory work done by Zilinsky et al. in which lidocaine and adrenaline did not alter the effects of TXA on the stability and fibrinolysis of blood clots (17).

### Limitations of the current study include

The current study has some limitations, including the lack of comparison with other surgical approaches like endoscopic DCR and the low number of patients.

## Conclusion

During external DCR using gauze soaked with combined TXA (100 mg/ml) and epinephrine 1:200,000 showed a significant reduction in the amount of intraoperative bleeding compared to gauze soaked with epinephrine 1:200,000 only. The reduction in the amount of bleeding with the addition of TXA results in a clearer surgical field, shorter surgical time, and more surgeon satisfaction.

## Data Availability

Data available on request from the authors.
